# Bladder perforation as a complication of transurethral resection of bladder tumors: the predictors, management, and its impact in a series of 1570 at a tertiary urology institute

**DOI:** 10.1007/s11255-023-03638-6

**Published:** 2023-06-15

**Authors:** Yasser Osman, Mohamed Elawdy, Diaa-Eldin Taha, Mohamed H. Zahran, Rasha T. Abouelkheir, Doaa Elsayed Sharaf, Ahmed Mosbah, Bedeir Ali-El Dein

**Affiliations:** 1 Urology Department, Urology and Nephrology Center, Mansoura University, Mansoura, Egypt; 2 Radiology Department, Urology and Nephrology Center, Mansoura University, Mansoura, Egypt; 3 Urology Departement, Faculty of Medicine, Kafr El-Sheikh University, Kafr El-Sheikh, Egypt; 4 Urology Department, Sohar Hospital, Ministry of Health, Sohar, Oman

**Keywords:** Bladder perforation, Transurethral resection of bladder tumors, TURB, TURBT, Bladder tumor, Management

## Abstract

**Objectives:**

To report the incidence, predictors, the impact of bladder perforation (BP), and our protocol of management in patients who underwent trans-urethral resection of bladder tumor (TURBT).

**Methods:**

This is a retrospective study, between 2006 and 2020, on patients who underwent TURBT for non-muscle-invasive bladder cancer (NMIBC). Bladder perforation was defined as any full thickness resection of the bladder wall. Bladder perforations were managed based on their severity and type. Small BP with no or mild symptoms were managed with prolongation of urethral catheters. Those with significant extraperitoneal extravasations were managed by insertion of a tube drain (TD). Abdominal exploration was done for extensive BP and all intraperitoneal extravasations.

**Results:**

Our study included 1,570 patients, the mean age was 58 ± 11 years and 86% were males. Bladder perforation was recorded in 10% (*n* = 158) of the patients. The perforation was extraperitoneal in 95%, and in 86%, the perforation was associated with no symptoms, mild symptoms, or mild fluid extravasation that required only prolongation of the urethral catheter. On the other hand, active intervention was required for the 21 remaining patients (14%) with TD being the most frequent management. History of previous TURBT (*p* = 0.001) and obturator jerk (*p* = 0.0001) were the only predictors for BP.

**Conclusions:**

The overall incidence of bladder perforation is 10%; however, 86% required only prolongation of urethral catheter. Bladder perforation did not affect the probability for tumor recurrence, tumor progression nor radical cystectomy.

## Introduction

Bladder tumors are the most common urothelial malignancies, and 75–80% are non-muscle invasive (NMIBC). Trans-urethral resection of bladder tumor (TURBT) is the primary surgical management for NMIBC and is considered one of the most common endourologic procedures [1]. It has been described as a reasonably safe procedure with bleeding and bladder perforation (BP) are the most common complications. Bleeding during TURBT can be controlled at the time of surgery, but BP is a special concern.

Bladder perforation has serious side effects, both in short- and long-term outcomes: BP could abort the procedure, preclude from giving intravesical therapy, and increase the risk of extravasation of tumor cells [2]. The literature has substantial reviews on the predictors of BP [3–5]; however, the impact of BP on bladder tumors was infrequently reported in the literature [4, 6].

In addition to reporting the incidence, predictors, and our protocol of management of BP, we also aimed to report on the impact of BP on bladder tumors outcome. This study included a relatively large series of patients at a single tertiary urology institute.

### Patients and methods

After obtaining the institutional review board approval (R.22.12.1975), a retrospective analysis was conducted on the hospital’s data base retrieving all patients who were diagnosed with non-muscle-invasive bladder cancer between January 2006 and December 2020. Patients who were treated with transurethral resection of bladder tumors (TURBT) were further analyzed.

Exclusion criteria: Patients with muscle-invasive bladder cancer, those with bladder tumors that had metastasized at the time of diagnosis, and those with non-cancerous tumors on the final histopathological reports were excluded from the study.

### Operative details

Spinal anesthesia was the preferred mode of anesthesia, and general anesthesia was used if the spinal anesthesia was contraindicated or failed. TURBT was done following the standard surgical principles. A single dose of intravesical chemotherapy epirubicin (50 mg) was given in the recovery room to eligible patients apparently with small-volume, low-grade NMIBC. Adjuvant intravesical chemoimmunotherapy was given based on the European guidelines protocol.

### Bladder perforation

Our study included not only symptomatic patients with obvious perforations that manifested with extravasation or abdominal distention, but also included silent perforation in asymptomatic patients. BP was defined as any full thickness resection of the bladder wall resulting in documented (gross) extravasation of irrigating fluid or recognition of perivesical fat intraoperatively. Bladder perforations were managed based on the time of recognition, types and its severity.

Intraoperatively, if minor extraperitoneal BP was discovered, the abdomen was checked, and if there were no signs of abdominal distention, the resection was to be performed quickly and hemostasis to be commenced as early as possible. However, if there were signs of abdominal distention, the procedure had to be terminated, and hemostasis was to be performed “if possible”.

Any major extraperitoneal bladder perforation with extensive extravasation and all intraperitoneal perforations were treated by abdominal exploration and the tears were repaired. Cystectomy was performed as an emergency procedure in select patients with a large burden of bladder tumors associated with extensive perforation, or uncontrolled bleeding.

If BP was associated with abdominal distention, the abdomen was screened by ultrasonography (US). No intervention was performed for those who had mild fluid collection limited to the pelvis. However, a moderate amount of extravasation with extension to the upper abdomen was managed by insertion of an abdominal tube drain (TD).

Postoperatively, minor extraperitoneal bladder perforation in asymptomatic patients was managed only by prolongation of the urethral catheter for 3–5 days. In major BP, abdominal TD was removed within 2–3 days and urethral catheter was kept for 7–14 based on the extent of the perforation. Catheters were removed at the outpatient clinic and cystogram was not a routine workup before catheter removal.

### The outcomes

The incidence of BP and the predictors were reported using bivariate and multivariate analyses, and the impact of BP on tumor recurrence and progression was studied. Additionally, our protocol in managing BP at a tertiary urology institute was documented in detail.

### Statistical analysis

The data were collected using IBM SPSS version 21 (IBM Corp., Armonk, NY, USA). For univariate analysis, frequency and percentage were used to express categorical variables. Mean and standard deviation were used to express the scale variables with normally distributed data. For bivariate analysis, Chi-square (χ ^2^) test was used for categorical variables. For scale variables, paired sample t-test was used for normally distributed data.

Multivariate analysis with a logistic regression model was generated for variables that yielded significance on bivariate analysis. In all tests, the significance was set at *p* < 0.05.

## Results

Of 1,592 patients with NMIBC who were managed by TURBT, 22 with incomplete files were eliminated from the analysis, leaving 1,570 patients eligible for analysis. The mean age was 58 ± 11 years, males outweighed females (86%), and the median follow up was 42 months (range:10–140). The vast majority of patients had staged T1, and nearly half of them were graded as GII bladder tumors. The other tumor characteristics are shown in Table 1.

**Table 1:** Patients’ demographic characteristics of 1570 patients who had TURBT for NMIBC

Variables	No	(%)
Age *(Mean* ± *SD)* BMI *(Mean* ± *SD)*	58 ± 11.5 28.2 ± 7.5	
Gender		
Male	1350	(86%)
Female	220	(14%)
Previous TURBT		
No	1162	(74)
Yes	408	(26)
Tumor size		
Less than 3 cm	820	(53)
3 cm or more	750	(47)
Tumor grade		
Grade I	188	(12)
Grade II	832	(53)
Grade III	550	(35)
Tumor stage		
Ta	377	(24)
T1	1083	(69)
Primary CIS	110	(7)
Number of previous TURBT		
De novo	1162	(74)
One recurrence	236	(15)
Two recurrence	94	(6)
Three recurrence	47	(3)
> Three recurrence	31	(2)
Tumor site (location)		
Posterior	346	(22)
Lateral walls	521	(33)
Anterior and domal	143	(9)
Trigone and BN	94	(6)
Multicentric	466	(30)
Tumor number		
Single	797	(51)
Multiple	773	(49)
Bladder perforation		
No	1412	(90)
Yes	158	(10)

**Fig. 1:**
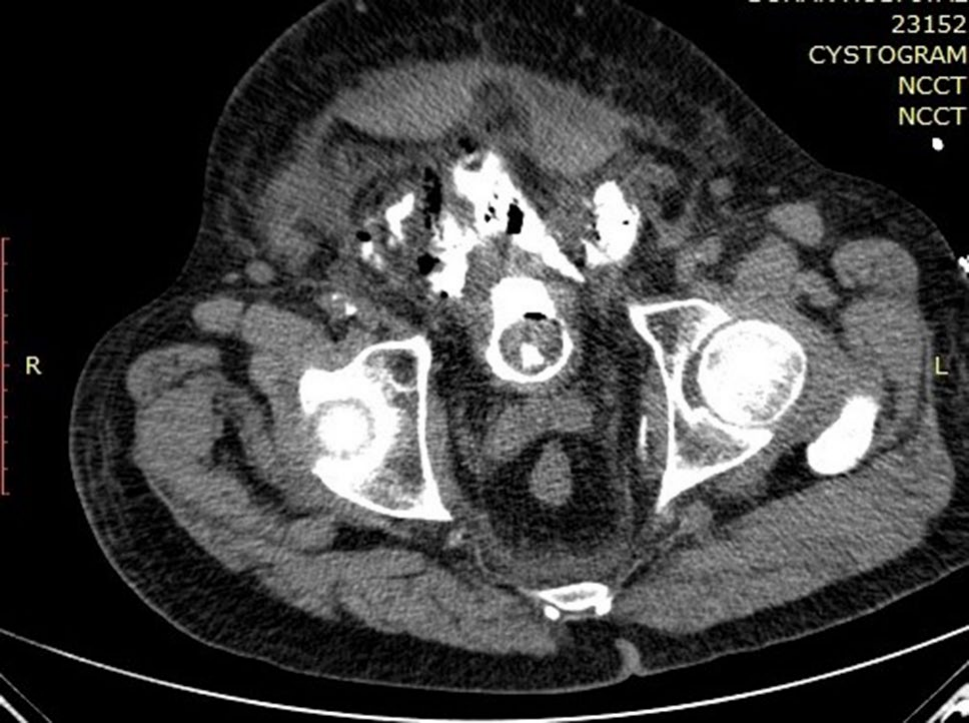
65-year-old male who came to the ER with an acute abdomen 5 days post TURBT and after 2 days of removal of the urethral catheter. CT cystogram axial view showing extravasation of the contrast outside the UB in the extraperitoneal space

**Fig. 2:**
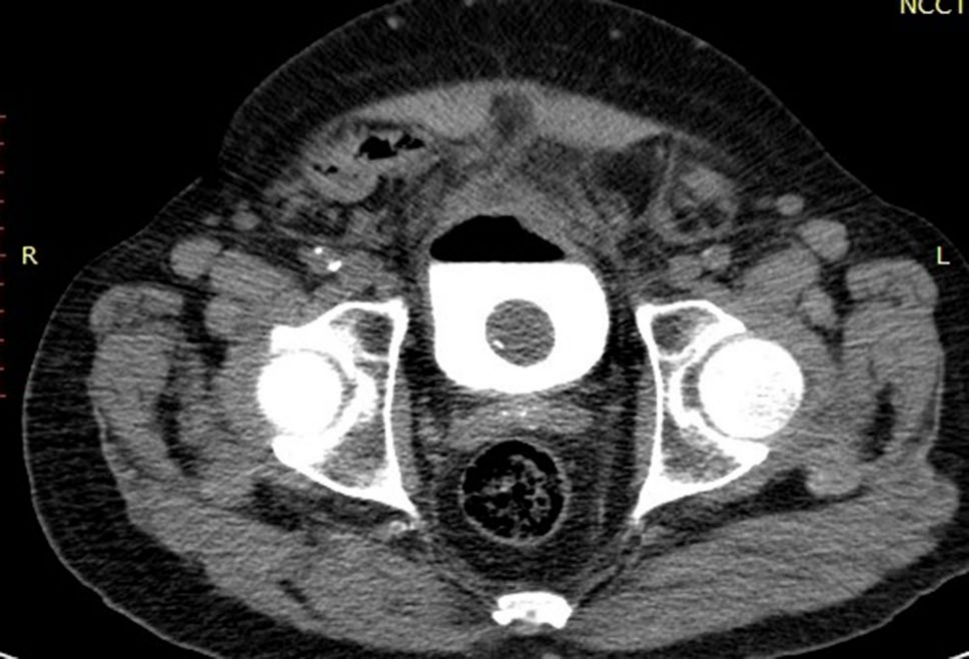
In the same patient, CT cystogram axial view with post indwelling urethral catheter for 10 days after the initial presentation showing the contrast in the UB with resolving of the extravasation

Bladder perforation was recorded in 10% (*n* = 158) of the patients, 95% of them were extraperitoneal (*n* = 149/158) and the rest were intraperitoneal. In the majority of the cases [86% (*n* = 137/158)], the perforation was associated with no symptoms, mild symptoms, or mild fluid extravasation that required only prolongation of the urethral catheter for a median of 5 (range, 3–10) days based on the extension of the BP with no other interventions, Figs. 1 and 2.

On the other hand, active intervention was required for the 21 remaining patients (14%); drain for 13, bladder repair in 6, and urgent cystectomy for only 2 patients.

On bivariate analysis, none of the following were predictors for BP: age, body mass index, gender, or tumor number/location. However, statistically significant predictors were the history of previous TURBT with the odds increasing as the number of previous TURBT increased (*p* = 0.001), tumor stage (*p* = 0.045), and occurrence of obturator jerk (*p* = 0.0001), Table 2.

**Table 2:** Bivariate and multivariate analysis of the possible predictors of perforation in patients with non-muscle-invasive bladder cancer (*N* = 1570)

Parameters	Bivariate analysis						Multivariate regression		
	Occurrence of bladder perforation				Odds ratio	*p*-value	Odds ratio	*95% CI*	*p*-value
	No N: 1412 (%)		Yes N: 158 (%)						
Gender									
Male	1219	(90)	131	(10)		0.2			
Female	193	(87)	27	(13)					
Previous TURBT									
No	1073	(92)	89	(8)	13	**0.001**	**2**	**1.4–**	**0.001**
Yes	339	(83)	69	(17)				**2.8**	
Number of previous TURBT									
De novo	1055	(91)	107	(9)					
One recurrence	205	(87)	31	(13)					
Two recurrences	88	(84)	6	(16)	19	**0.001**			0.08
Three recurrences	39	(84)	8	(16)					
> Three recurrence	25	(81)	6	(19)					
Tumor number									
Single	725	(91)	71	(9)	3.4	0.12			0.06
Multiple	687	(88)	87	(12)					
Tumor size									
Less than 3 cm	735	(89)	85	(11)					
3 cm or more	677	(90)	73	(10)		0.7			
Tumor grade									
Grade I	170	(91)	18	(9)					
Grade II	754	(91)	78	(9)		0.6			
Grade III	488	(89)	62	(11)					
Tumor stage									
Ta	354	(97)	23	(6)					
T1	956	(90)	127	(11)	7	**0.045**			
Primary CIS	102	(95)	8	(7)					
Tumor site (location)									
Posterior	315	(91)	31	(9)		0.08			
Lateral walls	459	(87)	62	(13)					
Anterior and domal	123	(86)	20	(14)					
Trigone and BN	86	(93)	8	(7)					
Multicentric	429	(92)	37	(8)					
Obturator jerk									
No	1404	(90)	149	(10)	30	**0.0001**	**10**	**3.8–**	**0.0001**
Yes	8	(47)	9	(53)				**27.2**	

Bold values indicate statistically significant values

On multivariate regression model, history of previous TURBT (OR = 2 [CI = 1.4–2.8], *p* = 0.001) and occurrence of obturator jerk (OR = 10, [CI = 3.8–27] *p* = 0.0001) sustained their significance, Table 2.

There were no statistical differences in the occurrence of bladder tumor recurrence (39/368 = 10.5% vs 119/1202 = 9.9%, *p* = 0.6), tumor progression (22/231 = 9.5% vs 29/1339 = 8.5%, *p* = 0.5), nor the probability for radical cystectomy (19/230 = 8.3% vs 139/1340 = 10.4%, *p* = 0.3) between bladder perforation vs non-perforation groups, respectively.

## Discussion

In our series, we recorded 10% incidence of BP, and the literature reported a wide range of BP incidence: 1–10% [4, 7, 8]. This wide range depends on the definition of BP, documentation, and the tools for diagnosis. We defined BP as any full thickness resection of the bladder wall resulting in documented (gross) extravasation of irrigating fluid or recognition of perivesical fat intraoperatively, and our protocol was to document any perforation, even in asymptomatic patients. For that reason, we recorded a quite higher incidence than those who reported only symptomatic BP. On the other hand, our incidence is considered low if compared to the series with an incidence of 50% that performed a routine postoperative cystogram, a method that discovers all tiny and trivial perforations which sometimes are not visible to surgeons [9]

Despite this wide range in the reporting of the incidence of BP, there is agreement in the literature that the majority of BP are extraperitoneal, mild and asymptomatic which can be treated only with prolongation of urethral catheters [8, 10, 11]. Similarly, in our research, 95% of BP were extraperitoneal, and 86% of the cases required only prolongation of the urethral catheter.

Bladder perforation can be identified at the time of resection, and the patient may have abdominal tenderness and distention, tachycardia, or low blood pressure that can be observed by the anesthesiologist. A high degree of suspicion is required by the surgeon to detect BP early as seen by a deep hole in the bladder wall and yellow, perivesical fat or inadequate bladder filling. In cases of very large defects with intraperitoneal extravasation, the surgeon may even notice inadequate bladder filling. In our series, and in most of the published series, the vast majority of the bladder tumors were in the posterior and lateral walls. Subsequently, it is expected that extraperitoneal perforation is more common than intraperitoneal perforation which we reported in our series at only 5% [12]. Anterior and domal bladder tumors were reported in only 9% of the entire series, and this could explain that fact. Bladder perforation could be accompanied by escape of irrigation fluids outside the bladder to the extraperitoneal space. With the escape of only a minimal amount of irrigation fluids, the problem is usually self-limited and does not require any active intervention. This occurred in 86% in our series, and there is agreement in the literature that BP does not require active intervention in the vast majority of patients [10, 12].

If the escape of fluid irrigation in the extraperitoneal space is quite large, Ultrasound can be used not only to diagnose fluid extravasation, but also can guide tube insertion for drainage. This method was the most common modality of intervention used in our series (60%). In contrast to the extraperitoneal space, the intraperitoneal space is open, and escape of irrigation fluid into this space is not self-limited and requires active intervention. Only 8 cases (5%) in our series required exploration, bladder repair was performed in six of them, and only two required urgent cystectomies. Most of the published series reported that exploration is the method of choice for intraperitoneal BP, however, insertion of a tube for drainage was reported infrequently in the literature [13] for intraperitoneal BP.

Factors that may predispose a patient to BP can be classified into four categories: patient, urinary bladder, tumor, and technical factors. Our analysis did not indicate a correlation between patient’s factors (such as age, gender and BMI) and the occurrence of BP, however, Herkommer et al. [7] reported female gender, and low BMI, and Golan et al. [5] reported elderly patients were predictors of BP occurrence. Bladder perforation was proved not to be due to deficient structure of the bladder wall itself. If the natural bladder wall was not a predictor, repeated resection could weaken the bladder wall due to fibrosis replacing the muscles and predispose the patient to BP.

In our series, we found that history of previous resection was an independent risk factor for the occurrence of BP (*p* = 0.001), with the odds increasing as the number of previous TURBT increased. This could be because bladder recurrence is more common on the tumor bed, and the same results were reported by [5] who found that BP is more likely to occur in heavily pretreated bladders.

Tumor characteristics could play a role in the occurrence of BP, and in our study, tumor stage was a predictor for BP on bivariate analysis, but it did not sustain its significance on multivariate analysis. However, tumor staging was reported by another study to be a predictor for BP (*p*-value = 0. 0.029)^8^. In that study, Herkommer et al. [7] included T2 bladder tumors that could be associated with deeper resections which increase the possibility of bladder perforation.

We could not find a correlation between tumor location, as reported by El Hayek et al. [9], where tumor location in the dome was a predisposing factor for bladder perforation.

No doubt that technical factors may play a role in the occurrence of BP. Obturator jerk (OJ) was an independent risk factor for BP in our study which sustained its significance in multivariate analysis (odds, 10 [CI = 3.8–27], *p* = 0.001). Because OJ was reported frequently in medical studies as a risk factor for BP [8, 14], researchers studied different surgical techniques to minimize the incidence of BP. Prospective studies and meta-analysis revealed that bipolar electrocautery has fewer complications than monopolar, but it is not superior to monopolar with respect to OJ and BP [15, 16]. On the other hand, there are differences reported between different types of bipolar Gyrus PlasmaKinetic system and Olympus TUR in saline (TURis) system [17]. Laser resection and en-block TUR resection using 980 nm laser fiber were reported to reduce the incidence of BP [18, 19]. Anesthetic techniques may play a role in the occurrence of OJ and BP. The results from systematic reviews on obturator nerve block reported that it lowered the obturator jerk (*p* = 0.001), and BP (*p* = 0.02) [14, 20].

Lee et al. [6] reported a few cases of tumor recurrence and progression after BP; however, the total cases of BP were 45 cases only, and the results were not solid to be compared. In our analysis, BP did not affect tumor recurrence, progression nor the probability of radical cystectomy. The impact of BP on survival was also infrequently reported in the literature. In a series that constituted 340 patients (7% = 26) who had BP, Skolarikos et al. [2] reported that open exploration after BP was an independent risk factor for extravesical recurrence and survival (*p* = 0.001). The possibility of microscopic tumor cell seeding on the bladder wall as a result of perforation could be the explanation; however, this matter requires more investigation. On the other hand, Golan et al. [5] reported that despite the potential comorbidities of BP, this adverse event does not seem to substantially increase the risk of extravesical tumor seeding.

It is to be mentioned that BP is a serious complication, and all measures are recommended to be taken to minimize its occurrence. Several measures proposed in the literature were focused on reduction of the likelihood of occurrence of obturator jerk, reducing the diathermy current, avoiding over-distention of the bladder, and laser use [8]. The presence or absence of detrusor muscle is a validated quality control indicator for the indicated cases (Tis & TaLG/G1-2 tumors, and TURBTs for random biopsies and re-TURBTs of scar excluded) [21]

The main limitation of our study is that it is a retrospective study. Additionally, TURBT were performed by different surgeons with varying levels of experience. Additionally, we do not evaluate time to recurrence, time to progression, and time to cystectomy. We did not include patients with T2 bladder tumor because it is a known risk factor for perforation.

Despite that, our study included a considerable number of patients who were treated at a single tertiary urology center. This provides insight in our protocol in managing bladder perforation in a relatively larger number of patients.

## Conclusion

We recorded a total of 10% incidence of bladder perforation with TURBT for NMIBC. History of previous bladder resection and obturator jerk were the only independent risk factors for the occurrence of BP. In the vast majority of patients (86%), the symptoms were mild and did not require any active interventions, but only prolongation of urethral catheter. Abdominal drainage was the most common procedure for those who required intervention. Bladder perforation did not affect bladder tumor recurrence, progression nor the probability for radical cystectomy.

## Data Availability

The data that support the findings of this study are available on request from the corresponding author [Mohamed Elawdy].
